# Supplementary material for: Characteristics of Floral Volatiles and Their Effects on Attracting Pollinating Insects in Three Bidens Species with Sympatric Distribution
Source: Biology (Basel). 2025 Sep 23;14(10):1310. doi: 10.3390/biology14101310 (PMC12561550; doi:10.3390/biology14101310)
Supplement: Supplementary file 1 [file biology-14-01310-s001.zip › biology-3859637-supplementary.docx]

**Supplementary Table S1:** Volatile components and relative content of flowers in three Bidens.

| **SN** | **sort** | **Name** | **CAS** | **Average relative content /% (mean±SD)** | | |
| --- | --- | --- | --- | --- | --- | --- |
|  |  |  |  | **BH** | **DL** | **SY** |
| 1 | alcohol | Ethanol,2-(methylamino)- | 109-83-1 | 2.58±2.82 | — | — |
| 2 | alcohol | (R)-(-)-2-Amino-1-propanol | 35320-23-1 | 2.16±0.79 | — | — |
| 3 | alcohol | Silanediol,dimethyl- | 1066-42-8 | 0.59±0.84 | — | — |
| 4 | alcohol | 1-Butanol,3-methyl- | 123-51-3 | — | — | 0.21±0.11 |
| 5 | alcohol | Bicyclo[3.1.1]hept-2-ene-2-methanol,6,6-dimethyl- | 20133-04-2 | 0.08±0.08 | — | — |
| 6 | alcohol | (+)-Cycloisolongifol-5-ol | 74841-81-9 | — | — | 0.05±0.05 |
| 7 | alcohol | p-Menthane-1,3-diol | 1612-98-2 | — | 0.05±0.05 | — |
| 8 | aromatic compound | Naphthalene,1,2,3,4,4a,5,6,8a-octahydro-7-methyl-4-methylene-1-(1-methylethyl)-,(1α,4aβ,8aα)- | 39029-41-9 | — | 0.1±0.12 | 0.28±0.15 |
| 9 | aromatic compound | Naphthalene,1,2,3,5,6,8a-hexahydro-4,7-dimethyl-1-(1-methylethyl)-,(1S-cis)- | 483-76-1 | — | 0.09±0.09 | 0.28±0.16 |
| 10 | aromatic compound | Naphthalene,1,2,3,4,4a,5,6,8a-octahydro-4a,8-dimethyl-2-(1-methylethenyl)-,[2R-(2α,4aα,8aβ)]- | 38872-77-8 | — | 0.03±0.03 | — |
| 11 | aromatic compound | 2,5,5,8a-Tetramethyl-3,4,4a,5,6,8a-hexahydro-2H-chromene | * | — | — | 0.02±0.02 |
| 12 | aromatic compound | p-(1-Propenyl)-toluene | 2077-30-7 | — | 0.16±0.05 | — |
| 13 | aromatic compound | Naphthalene,decahydro-4a-methyl-1-methylene-7-(1-methylethenyl)-,[4aR-(4aα,7α,8aβ)]- | 17066-67-0 | — | 0.07±0.04 | — |
| 14 | aromatic compound | 4a,8-Dimethyl-2-(prop-1-en-2-yl)-1,2,3,4,4a,5,6,7-octahydronaphthalene | 103827-22-1 | — | 0.05±0.07 | — |
| 15 | terpene | (+)-α-Pinene | 7785-70-8 | — | — | 27.19±24.18 |
| 16 | terpene | (Z)-β-Ocimene | 13877-91-3 | 33.93±3.49 | 1.49±0.44 | 0.13±0.05 |
| 17 | terpene | α-Muurolene | 483-75-0 | 0.4±0.47 | 0.06±0.02 | 3.28±2.47 |
| 18 | terpene | (E)-β-Ocimene | 3779-61-1 | 18.31±1.1 | 0.23±0.06 | 0.19±0.14 |
| 19 | terpene | D-Limonene | 5989-27-5 | 5.87±4.45 | — | — |
| 20 | terpene | α-Phellandrene | 99-83-2 | — | 11.17±0.99 | — |
| 21 | terpene | β-Myrcene | 123-35-3 | 3.25±3.22 | 11.97±1.00 | 2.54±0.62 |
| 22 | terpene | α-Pinene | 80-56-8 | 8.67±1.47 | 8.41±0.41 | — |
| 23 | terpene | Neo-alloocimene | 7216-56-0 | 9.91±0.58 | 0.09±0.04 | — |
| 24 | terpene | 3-Carene | 13466-78-9 | — | — | 3.59±1.35 |
| 25 | terpene | Caryophyllene | 87-44-5 | 1.1±1.13 | 0.45±0.09 | 2.9±1.03 |
| 26 | terpene | Camphene | 79-92-5 | 0.19±0.06 | 0.81±0.17 | 0.2±0.06 |
| 27 | terpene | GermacreneD | 23986-74-5 | — | — | 0.69±0.45 |
| 28 | terpene | .gamma.-Terpinene | 99-85-4 | 0.09±0.05 | 0.18±0.02 | — |
| 29 | terpene | Humulene | 6753-98-6 | 0.15±0.11 | 0.1±0.04 | 0.43±0.32 |
| 30 | terpene | trans-α-Bergamotene | 13474-59-4 | 0.09±0.07 | 0.18±0.05 | — |
| 31 | terpene | 1-Octene | 111-66-0 | 0.01±0.02 | 0.04±0.04 | — |
| 32 | terpene | .gamma.-Muurolene | 30021-74-0 | 0.04±0.06 | — | — |
| 33 | terpene | (-)-β-Bourbonene | 4547-24-4 | 0.05±0.05 | — | — |
| 34 | terpene | α-Cubebene | 17699-14-8 | 0.08±0.13 | 0.06±0.02 | 0.25±0.19 |
| 35 | terpene | Copaene | 3856-25-5 | 0.2±0.14 | 0.18±0.04 | 0.4±0.3 |
| 36 | terpene | Cyclohexene,3-methyl-6-(1-methylethylidene)- | 586-62-9 | — | — | 0.07±0.07 |
| 37 | terpene | Pinocarvone | * | 0.06±0.02 | — | — |
| 38 | terpene | Sabinene | 3387-41-5 | — | — | 35.02±6.79 |
| 39 | terpene | Caryophylleneoxide | 1139-30-6 | 0.05±0.05 | — | — |
| 40 | terpene | m-Cymene | 535-77-3 | — | 52.23±1.9 | — |
| 41 | ketone | Bicyclo[3.1.1]heptan-3-one,2,6,6-trimethyl-,(1α,2β,5α)- | 14575-93-0 | — | — | 0.08±0.07 |
| 42 | ketone | Bicyclo[3.1.1]hept-3-en-2-one,4,6,6-trimethyl- | 18309-32-5 | — | — | 0.02±0.04 |
| 43 | ketone | Thujone | 546-80-5 | — | — | 0.03±0.02 |
| 44 | ketone | Bicyclo[3.1.1]heptan-3-one,2,6,6-trimethyl-,(1α,2α,5α)- | 15358-88-0 | 0.02±0.02 | — | 0.02±0.03 |
| 45 | alkane | Bicyclo[3.1.1]heptane,6,6-dimethyl-2-methylene-,(1S)- | 366-18-7 | 0.8±0.11 | 1.05±0.13 | 2.76±2.53 |
| 46 | alkane | Cyclohexane,1-methylene-4-(1-methylethenyl)- | 499-97-8 | — | — | 2.32±0.39 |
| 47 | alkane | (1R,2S,6S,7S,8S)-8-Isopropyl-1-methyl-3-methylenetricyclo[4.4.0.02,7]decane-rel- | 18252-44-3 | 0.9±0.87 | 0.02±0.02 | 0.52±0.46 |
| 48 | alkane | Tridecane,2-methyl- | 1560-96-9 | — | — | 0.05±0.06 |
| 49 | alkane | Tridecane | 629-50-5 | — | — | 0.04±0.04 |
| 50 | alkane | Cyclohexane,1-ethenyl-1-methyl-2,4-bis(1-methylethenyl)-,[1S-(1α,2β,4β)]- | 515-13-9 | — | 0.05±0.05 | 0.09±0.06 |
| 51 | alkene | Cyclohexene,1-methyl-5-(1-methylethenyl)-,(R)- | 13898-73-2 | — | 2.41±0.09 | — |
| 52 | alkene | (E)-4,8-Dimethylnona-1,3,7-triene | 19945-61-0 | 2.08±1.17 | 0.27±0.09 | 1.35±0.67 |
| 53 | alkene | 1,3-Cyclohexadiene,1-methyl-4-(1-methylethyl)- | 99-86-5 | 0.01±0.01 | 0.31±0.04 | 0.35±0.06 |
| 54 | alkene | 2,6-Dimethyl-1,3,5,7-octatetraene,E,E- | 460-01-5 | 0.18±0.03 | — | — |
| 55 | alkene | cis-Muurola-4(15),5-diene | 157477-72-0 | — | 0.02±0.02 | 0.13±0.1 |
| 56 | alkene | Bicyclo[3.1.0]hex-2-ene,2-methyl-5-(1-methylethyl)- | 2867/5/2 | 0.01±0.01 | 0.11±0.02 | 0.22±0.05 |
| 57 | alkene | Cyclohexene,1-methyl-4-(1-methylethylidene)- | 586-62-9 | 0.03±0.03 | 0.09±0.01 | — |
| 58 | alkene | 1,4-Cyclohexadiene,3-ethenyl-1,2-dimethyl- | 62338-57-2 | 0.13±0.09 | — | 0.05±0.09 |
|  |  |  |  |  |  |  |
|  |  |  |  |  |  |  |
| 59 | ester | Bornylacetate | 76-49-3 | — | 1.23±0.03 | — |
| 60 | ester | Myrtenylacetate | 2623-23-6 | 0.31±0.28 | — | — |
| 61 | ester | Butanoicacid,2-methyl-,1-methylpropylester | 869-08-9 | 0.1±0.09 | — | — |
| 62 | ester | Propanoicacid,2-methyl-,3-hexenylester,(E)- | * | 0.18±0.19 | — | — |
| 63 | ester | Cyclopentanecarboxylicacid,undecylester | * | 0.14±0.09 | — | — |
| 64 | ester | Cyclopentanecarboxylicacid,tridecylester | * | 0.06±0.06 | — | — |

Note : " * " means CAS number of the corresponding substance was not found, and " — " means that it was not detected.
